# 2-(4-Chloro­phen­yl)-3-*p*-tolyl-1,3-thia­zolidin-4-one

**DOI:** 10.1107/S1600536809010307

**Published:** 2009-03-25

**Authors:** Xiao-Jun Sun, Jian-Feng Zhou, Zai-Chao Zhang, Yu-Jie Wang

**Affiliations:** a Department of Chemistry, Huaiyin Teachers College, Huaian 223001, People’s Republic of China

## Abstract

The title compound, C_16_H_14_ClNOS, a potent anti­bacterial chemical, features a dihedral angle of 49.4 (1)° between the 4-tolyl and thia­zolidinone rings, and a dihedral angle of 87.2 (5)° between the thia­zolidinone and 4-chloro­phenyl rings.

## Related literature

For the synthesis, see: Srivastava *et al.* (2002 ▶).
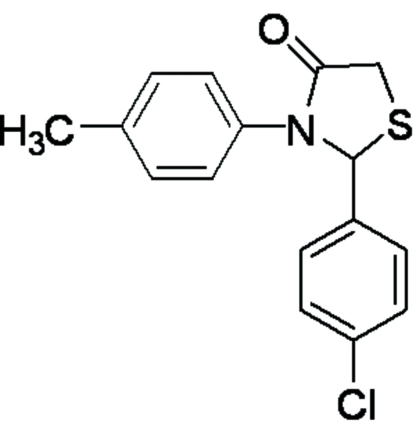


## Experimental

### 

#### Crystal data

C_16_H_14_ClNOS
*M* *_r_* = 303.79
Orthorhombic, 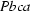

*a* = 12.1591 (4) Å
*b* = 13.0708 (4) Å
*c* = 18.5125 (7) Å
*V* = 2942.18 (17) Å^3^
*Z* = 8
Mo *K*α radiation
μ = 0.40 mm^−1^
*T* = 296 K
0.40 × 0.35 × 0.20 mm

#### Data collection

Bruker APEXII area-detector diffractometer
Absorption correction: multi-scan (*SADABS*; Bruker, 2000 ▶) *T*_min_ = 0.85, *T*_max_ = 0.92
16959 measured reflections
3377 independent reflections
2205 reflections with *I* > 2˘*I*)
*R*_int_ = 0.058

#### Refinement

*R*[*F* ^2^ > 2σ(*F* ^2^)] = 0.044
*wR*(*F* ^2^) = 0.115
*S* = 1.02
3377 reflections
182 parameters
H-atom parameters constrained
Δρ_max_ = 0.23 e Å^−3^
Δρ_min_ = −0.34 e Å^−3^

### 

Data collection: *APEX2* (Bruker, 2004 ▶); cell refinement: *SAINT* (Bruker, 2004 ▶); data reduction: *SAINT*; program(s) used to solve structure: *SHELXS97* (Sheldrick, 2008 ▶); program(s) used to refine structure: *SHELXL97* (Sheldrick, 2008 ▶); molecular graphics: *ORTEP-3 for Windows* (Farrugia, 1997 ▶); software used to prepare material for publication: *SHELXL97* and *PLATON* (Spek, 2009 ▶).

## Supplementary Material

Crystal structure: contains datablocks global, I. DOI: 10.1107/S1600536809010307/ng2565sup1.cif

Structure factors: contains datablocks I. DOI: 10.1107/S1600536809010307/ng2565Isup2.hkl

Additional supplementary materials:  crystallographic information; 3D view; checkCIF report

## Figures and Tables

**Fig. 1.**
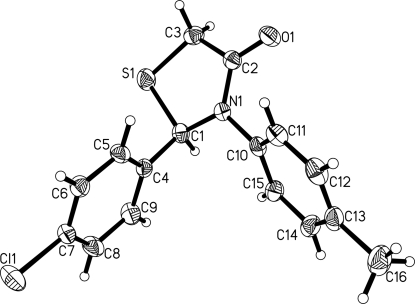
The molecular structure of (I), showing the atom-numbering schem. Displacement ellipsoids are drawn at the 30% probability level.

## supplementary crystallographic information

### Comment

4-thiazolidinone ring system comprises the broad spectrum for a number of biologically active compounds.In recent years, 4-thiazolidinones are the most extensively investigated class of compounds, which exhibits various biological activities, such as anticancer, antitubercular, antibacterial and herbicidal activities. In view of these properties and in a continuation of our interest in the chemistry of 4-thiazolidinones, we have attempted to synthesize a series of 4-thiazolidinone derivatives, some of which have comparatively high antibacterial activity. The crystal structure determination of the title compound,(I), was undertaken to investigate the relationship between structure and antibacterial activity(Fig. 1). The molecular conformation is described by the dihedral angle between 4-methylbenzene ring and thiazolidinone ring of 49.4 (1)° and the dihedral angle between thiazolidinone ring and 4-chlorobenzene ring of 87.2 (5)°.

### Experimental

Compound (I) was synthesized according to the procedure of Tumul Srivastava *et al.* (2002). A crystal of (I) suitable for X-ray analysis was grown from an ethanol solution by slow evaporation at room temperature.

### Refinement

H atoms were placed in idealized positions and constrained to ride on their parent atoms, with C—H distances of 0.95 (aromatic), 0.99 (methylene), 1.00 (methylidyne) and 0.98 Å(methyl), and with *U*_iso_(H) = 1.2*U*_eq_(C) or 1.5*U*_eq_(C_methyl_).

### Crystal data

**Table:**

C_16_H_14_ClNOS	*F*(000) = 1264
*M**_r_* = 303.79	*D*_x_ = 1.372 Mg m^−^^3^
Orthorhombic, *P**b**c**a*	Mo *K*α radiation, λ = 0.71073 Å
Hall symbol: -P 2ac 2ab	Cell parameters from 2211 reflections
*a* = 12.1591 (4) Å	θ = 2.5–25.0°
*b* = 13.0708 (4) Å	µ = 0.40 mm^−^^1^
*c* = 18.5125 (7) Å	*T* = 296 K
*V* = 2942.18 (17) Å^3^	Plate, colorless
*Z* = 8	0.40 × 0.35 × 0.20 mm

### Data collection

**Table:**

Bruker APEXII area-detector diffractometer	3377 independent reflections
Radiation source: fine-focus sealed tube	2205 reflections with *I* > 2˘*I*)
graphite	*R*_int_ = 0.058
Detector resolution: 0 pixels mm^-1^	θ_max_ = 27.5°, θ_min_ = 2.2°
w\ scans	*h* = −14→15
Absorption correction: multi-scan (*SADABS*; Bruker, 2000)	*k* = −16→16
*T*_min_ = 0.85, *T*_max_ = 0.92	*l* = −24→24
16959 measured reflections	

### Refinement

**Table:**

Refinement on *F*^2^	Primary atom site location: structure-invariant direct methods
Least-squares matrix: full	Secondary atom site location: difference Fourier map
*R*[*F*^2^ > 2σ(*F*^2^)] = 0.044	Hydrogen site location: inferred from neighbouring sites
*wR*(*F*^2^) = 0.115	H-atom parameters constrained
*S* = 1.02	*w* = 1/[σ^2^(*F*_o_^2^) + (0.0467*P*)^2^ + 0.6312*P*] where *P* = (*F*_o_^2^ + 2*F*_c_^2^)/3
3377 reflections	(Δ/σ)_max_ = 0.002
182 parameters	Δρ_max_ = 0.23 e Å^−^^3^
0 restraints	Δρ_min_ = −0.34 e Å^−^^3^

### Special details

**Table:**

Geometry. All e.s.d.'s (except the e.s.d. in the dihedral angle between two l.s. planes) are estimated using the full covariance matrix. The cell e.s.d.'s are taken into account individually in the estimation of e.s.d.'s in distances, angles and torsion angles; correlations between e.s.d.'s in cell parameters are only used when they are defined by crystal symmetry. An approximate (isotropic) treatment of cell e.s.d.'s is used for estimating e.s.d.'s involving l.s. planes.
Refinement. Refinement of *F*^2^ against ALL reflections. The weighted *R*-factor *wR* and goodness of fit *S* are based on *F*^2^, conventional *R*-factors *R* are based on *F*, with *F* set to zero for negative *F*^2^. The threshold expression of *F*^2^ > σ(*F*^2^) is used only for calculating *R*-factors(gt) *etc*. and is not relevant to the choice of reflections for refinement. *R*-factors based on *F*^2^ are statistically about twice as large as those based on *F*, and *R*- factors based on ALL data will be even larger.

### Fractional atomic coordinates and isotropic or equivalent isotropic displacement parameters (Å^2^)

**Table:**

	*x*	*y*	*z*	*U*_iso_*/*U*_eq_	
C1	0.66878 (17)	0.93240 (14)	0.34463 (11)	0.0402 (5)	
H1	0.6105	0.9535	0.3112	0.048*	
C2	0.85314 (18)	0.96718 (14)	0.30086 (12)	0.0399 (5)	
C3	0.83681 (18)	1.05986 (15)	0.34764 (13)	0.0467 (6)	
H3A	0.8310	1.1207	0.3179	0.056*	
H3B	0.8990	1.0681	0.3800	0.056*	
C4	0.62581 (16)	0.85053 (14)	0.39467 (11)	0.0368 (5)	
C5	0.69556 (17)	0.79865 (16)	0.44122 (12)	0.0434 (5)	
H5	0.7708	0.8112	0.4391	0.052*	
C6	0.65582 (18)	0.72901 (15)	0.49046 (12)	0.0441 (5)	
H6	0.7033	0.6949	0.5217	0.053*	
C7	0.54441 (18)	0.71086 (15)	0.49257 (12)	0.0429 (5)	
C8	0.47397 (18)	0.75886 (19)	0.44628 (14)	0.0546 (6)	
H8	0.3990	0.7448	0.4478	0.065*	
C9	0.51497 (18)	0.82863 (18)	0.39704 (13)	0.0508 (6)	
H9	0.4673	0.8611	0.3652	0.061*	
C10	0.76107 (17)	0.81131 (14)	0.25927 (11)	0.0380 (5)	
C11	0.84795 (18)	0.74324 (15)	0.25858 (13)	0.0468 (5)	
H11	0.9090	0.7546	0.2877	0.056*	
C12	0.8436 (2)	0.65774 (16)	0.21407 (14)	0.0552 (6)	
H12	0.9030	0.6130	0.2130	0.066*	
C13	0.7532 (2)	0.63756 (16)	0.17140 (12)	0.0519 (6)	
C14	0.6660 (2)	0.70546 (16)	0.17427 (12)	0.0510 (6)	
H14	0.6037	0.6929	0.1465	0.061*	
C15	0.66950 (18)	0.79175 (15)	0.21761 (12)	0.0433 (5)	
H15	0.6101	0.8365	0.2186	0.052*	
C16	0.7492 (3)	0.54493 (19)	0.12222 (16)	0.0780 (9)	
H16A	0.8176	0.5085	0.1252	0.117*	
H16B	0.7372	0.5668	0.0733	0.117*	
H16C	0.6902	0.5008	0.1370	0.117*	
Cl1	0.49492 (5)	0.62309 (5)	0.55527 (4)	0.0680 (2)	
N1	0.76603 (13)	0.90125 (11)	0.30369 (9)	0.0371 (4)	
O1	0.93527 (13)	0.95428 (11)	0.26448 (9)	0.0529 (4)	
S1	0.71335 (6)	1.04265 (4)	0.39863 (3)	0.0568 (2)	

### Atomic displacement parameters (Å^2^)

**Table:**

	*U*^11^	*U*^22^	*U*^33^	*U*^12^	*U*^13^	*U*^23^
C1	0.0406 (12)	0.0425 (11)	0.0373 (12)	0.0056 (9)	0.0029 (10)	0.0017 (9)
C2	0.0395 (12)	0.0439 (11)	0.0364 (12)	0.0001 (9)	−0.0021 (10)	0.0069 (9)
C3	0.0489 (13)	0.0427 (11)	0.0484 (14)	−0.0029 (9)	−0.0055 (11)	0.0022 (10)
C4	0.0358 (11)	0.0427 (10)	0.0319 (11)	0.0016 (8)	0.0025 (9)	−0.0025 (9)
C5	0.0305 (11)	0.0568 (12)	0.0429 (13)	−0.0040 (9)	−0.0001 (10)	0.0055 (10)
C6	0.0405 (13)	0.0533 (12)	0.0385 (13)	0.0006 (9)	−0.0027 (10)	0.0038 (10)
C7	0.0431 (13)	0.0470 (11)	0.0385 (12)	−0.0054 (9)	0.0083 (10)	−0.0005 (9)
C8	0.0306 (12)	0.0760 (15)	0.0571 (16)	−0.0067 (10)	0.0021 (11)	0.0084 (13)
C9	0.0403 (13)	0.0668 (14)	0.0452 (14)	0.0062 (10)	−0.0020 (11)	0.0077 (12)
C10	0.0431 (12)	0.0387 (10)	0.0322 (11)	−0.0010 (8)	0.0073 (10)	0.0046 (8)
C11	0.0419 (13)	0.0472 (11)	0.0512 (15)	0.0024 (9)	0.0064 (11)	0.0056 (10)
C12	0.0616 (16)	0.0423 (11)	0.0616 (17)	0.0112 (10)	0.0206 (14)	0.0076 (11)
C13	0.0736 (17)	0.0411 (11)	0.0409 (13)	−0.0031 (11)	0.0150 (13)	0.0008 (10)
C14	0.0659 (16)	0.0492 (12)	0.0379 (13)	−0.0041 (11)	−0.0044 (12)	0.0001 (10)
C15	0.0489 (13)	0.0429 (11)	0.0381 (12)	0.0045 (9)	−0.0015 (10)	0.0013 (9)
C16	0.120 (2)	0.0507 (14)	0.0634 (18)	0.0008 (15)	0.0190 (18)	−0.0083 (13)
Cl1	0.0592 (4)	0.0747 (4)	0.0701 (5)	−0.0107 (3)	0.0135 (3)	0.0234 (4)
N1	0.0368 (9)	0.0405 (8)	0.0339 (9)	−0.0006 (7)	0.0038 (8)	−0.0005 (7)
O1	0.0424 (9)	0.0556 (9)	0.0606 (11)	−0.0043 (7)	0.0109 (8)	0.0000 (8)
S1	0.0723 (5)	0.0469 (3)	0.0513 (4)	−0.0062 (3)	0.0169 (3)	−0.0087 (3)

### Geometric parameters (Å, °)

**Table:**

C1—N1	1.462 (2)	C8—C9	1.382 (3)
C1—C4	1.509 (3)	C8—H8	0.9300
C1—S1	1.836 (2)	C9—H9	0.9300
C1—H1	0.9800	C10—C15	1.378 (3)
C2—O1	1.216 (2)	C10—C11	1.381 (3)
C2—N1	1.366 (3)	C10—N1	1.436 (2)
C2—C3	1.502 (3)	C11—C12	1.390 (3)
C3—S1	1.787 (2)	C11—H11	0.9300
C3—H3A	0.9700	C12—C13	1.378 (3)
C3—H3B	0.9700	C12—H12	0.9300
C4—C9	1.378 (3)	C13—C14	1.384 (3)
C4—C5	1.386 (3)	C13—C16	1.516 (3)
C5—C6	1.376 (3)	C14—C15	1.385 (3)
C5—H5	0.9300	C14—H14	0.9300
C6—C7	1.376 (3)	C15—H15	0.9300
C6—H6	0.9300	C16—H16A	0.9600
C7—C8	1.364 (3)	C16—H16B	0.9600
C7—Cl1	1.740 (2)	C16—H16C	0.9600
			
N1—C1—C4	113.63 (15)	C4—C9—H9	119.7
N1—C1—S1	105.19 (13)	C8—C9—H9	119.7
C4—C1—S1	108.94 (14)	C15—C10—C11	119.55 (19)
N1—C1—H1	109.6	C15—C10—N1	120.41 (18)
C4—C1—H1	109.6	C11—C10—N1	120.04 (19)
S1—C1—H1	109.6	C10—C11—C12	119.6 (2)
O1—C2—N1	124.76 (19)	C10—C11—H11	120.2
O1—C2—C3	122.66 (19)	C12—C11—H11	120.2
N1—C2—C3	112.58 (19)	C13—C12—C11	121.6 (2)
C2—C3—S1	108.30 (14)	C13—C12—H12	119.2
C2—C3—H3A	110.0	C11—C12—H12	119.2
S1—C3—H3A	110.0	C12—C13—C14	117.8 (2)
C2—C3—H3B	110.0	C12—C13—C16	121.6 (2)
S1—C3—H3B	110.0	C14—C13—C16	120.7 (3)
H3A—C3—H3B	108.4	C13—C14—C15	121.4 (2)
C9—C4—C5	118.47 (19)	C13—C14—H14	119.3
C9—C4—C1	120.36 (19)	C15—C14—H14	119.3
C5—C4—C1	121.12 (18)	C10—C15—C14	120.0 (2)
C6—C5—C4	121.38 (19)	C10—C15—H15	120.0
C6—C5—H5	119.3	C14—C15—H15	120.0
C4—C5—H5	119.3	C13—C16—H16A	109.5
C5—C6—C7	118.6 (2)	C13—C16—H16B	109.5
C5—C6—H6	120.7	H16A—C16—H16B	109.5
C7—C6—H6	120.7	C13—C16—H16C	109.5
C8—C7—C6	121.4 (2)	H16A—C16—H16C	109.5
C8—C7—Cl1	120.35 (17)	H16B—C16—H16C	109.5
C6—C7—Cl1	118.25 (17)	C2—N1—C10	121.80 (17)
C7—C8—C9	119.4 (2)	C2—N1—C1	118.11 (16)
C7—C8—H8	120.3	C10—N1—C1	119.39 (15)
C9—C8—H8	120.3	C3—S1—C1	93.39 (9)
C4—C9—C8	120.7 (2)		
